# Comparison of Predictive Control Methods for High Consumption Industrial Furnace

**DOI:** 10.1155/2013/279042

**Published:** 2013-11-10

**Authors:** Goran Stojanovski, Mile Stankovski

**Affiliations:** Department of Automation and System Engineering, Faculty of Electrical Engineering and Information Technologies, Saints Cyril and Methodius University in Skopje, ul. Ruger Boskovic bb., 1000 Skopje, Macedonia

## Abstract

We describe several predictive control approaches for high consumption industrial furnace control. These furnaces are major consumers in production industries, and reducing their fuel consumption and optimizing the quality of the products is one of the most important engineer tasks. In order to demonstrate the benefits from implementation of the advanced predictive control algorithms, we have compared several major criteria for furnace control. On the basis of the analysis, some important conclusions have been drawn.

## 1. Introduction

Processes and plant constructions of thermal systems and industrial furnaces, kilns and ovens in particular, have been subject to both scientific and technological research for long time [1]. This is mainly due to the process complexity of energy conversion and transfer in thermal systems. However, their control and supervision have recently become topics of extensive research due to the increased computing power of the controllers.

The overall control task in thermal processes is to drive the process to the desired thermodynamic equilibrium and to regulate the temperature profile through the plant. In industrial operating environment, technical control specifications involve goal and task descriptions of aims and procedures of supervision functions. From the general systems theoretical standpoint, it is the thermal systems where it became apparent that controlled processes in the real-world plants constitute a nonseparable, unique interplay of the three fundamental natural quantities: energy, mass, and information. If the stability problem is resolved, in all thermal processes the controller must optimize between the low consumption and the quality of the products [2, 3]. This compromise could be made by an experienced engineer or by an automated program that can optimize the behavior of the whole process. Thus, in recent years, we witness rapid utilization of control optimization techniques in order to improve the efficiency of the power plants. Since the technology has not evolved very much in the recent years, these goals are mainly achieved through the design of advanced control algorithms.

One of the most used optimization techniques in industry is the model predictive control (MPC). This algorithm utilizes the model of a plant in order to perform iterative predictions and optimize the control actions over some defined horizon. The effectiveness of this method directly depends on the quality of the model that represents the system. On the other hand, the implementation of linear MPC algorithms is straightforward, but implementation of MPC based on complex nonlinear models is still a topic of extensive research. Different computational methods for MPC implementation have been proposed in the recent years [4]. Most of them are based on nonlinear or hybrid models, but a generalization of the characteristics cannot be made, because every system has its own specifics, and must be considered separately.

The conventional control methods for high consumption industrial furnaces generally use linearized models [5–7] of the plants near the operation point, but very often these plants can be used for production of different types of products; hence, multiple operating points are required. The standard MPC algorithms do not provide an efficient solution to this problem. That is why the engineers turn towards utilization of switched or hybrid MPC algorithms. In this paper, we will explain the need for the implementation of switched and hybrid algorithms for the control of high consumption industrial furnaces.

Here, we present several predictive control algorithms that will be used for the optimization of high consumption (20 MW) industrial furnace. These algorithms will predict the system behavior on the basis of several models of the furnace. The original model of the furnace is derived using contemporary identification methods in [8]. Nevertheless, in this research we needed to improve the model and to enrich it with the variables that were ignored but have crucial impact on the process dynamics. Here, we explain the detailed technical description of the furnace and the process of building hybrid model that will be used for the design of hybrid MPC [9].

### 1.1. Constraints and Performance Criteria

**Figure 1:**
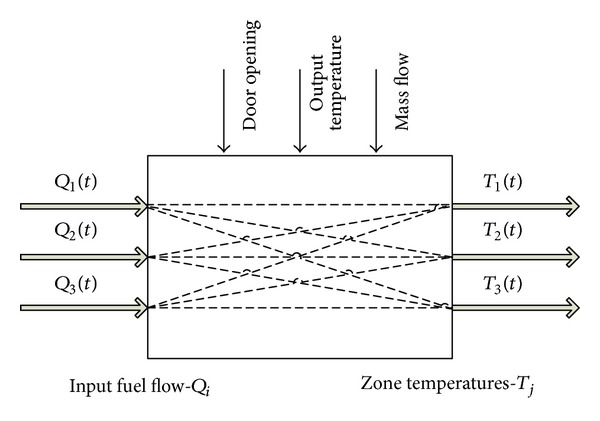
Diagram of the conceptual MIMO system model for gas-fired furnace in FZC “11 Oktomvri.”

Before we introduce the hybrid model, we need to elaborate the furnace dynamics. In this paper, we are dealing with 3-input 3-output gas-fired furnace as presented in Figure 1. The maximum temperature that can be achieved is 1150°Celsius when operating at full power (the valves for the burners are 100% open). 

The furnace has two openings (hatches) located at the front and the back of the furnace. When a pipe is entering the furnace, the front hatch must be opened. Logically, when there is a pipe exiting from the furnace, the back hatch must be opened. The opening of these hatches introduces a significant deviation (decreasing) of the temperature near the hatch. Since the front hatch is located near zone 1 of the furnace and the back hatch near zone 3 of the furnace, and using some experimental results, we can define the influence of the hatch opening to the global temperature throughout the three zones of the furnace. Considering these discrete changes in the behavior of the furnace and the nonlinearity in the continual domain, we can conclude that linearizing the furnace in one operating point is not acceptable for designing of a model predictive controller. 

For that reason, several operating points of the furnace are adopted. In this case, the linearization will be done in the surroundings of 130, 390, 650, 910, and 1170 degrees Celsius. If we chose to represent the rules for logic switching in the first zone of the furnace, for only one of the previously defined linearization points, then we can define a discrete automaton for the mode selection. 

### 1.2. Design of a Hybrid Model of the Furnace

It is obvious that the system is both discrete and nonlinear by nature but cannot be implemented as a discrete control system because of the logical conditions in the transfer function and the interconnection between the states and variables that combine a nonaffine set for synthesis of the control system.

In this paper, we define a hybrid model of the furnace that will incorporate the influence on both continuous and discrete dynamics, with additional consideration for logic and integer variables. As one of the emerging modeling languages, HYSDEL will be used to model the industrial furnace.

**Figure 2:**
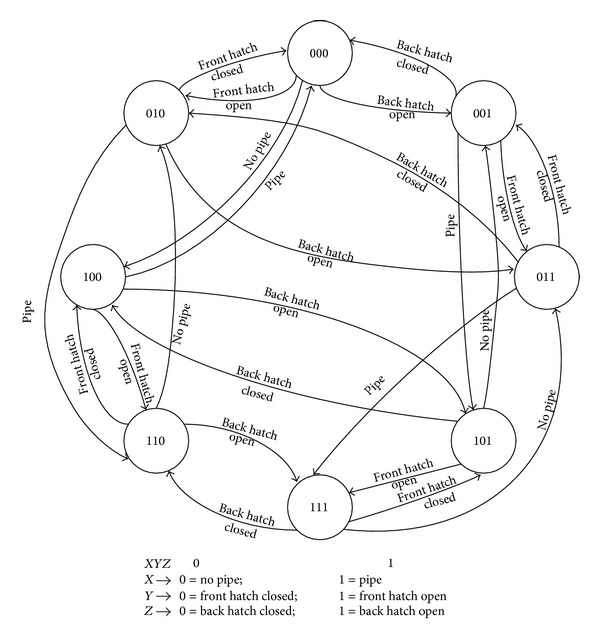
Discrete automaton for state switching in the first zone of the industrial furnace [9].

Considering that there are only 3 Boolean variables, the automaton will have 8 states. In Figure 2, we present the discrete automaton for the first zone of the furnace. Similar discrete automatons are designed for the second and for the third zones.

Of course here we do not represent the state changes when the furnace is crossing the border between one and another linearized model. In the discrete hybrid model, we can define these switches as logic conditions (state 1_3 = *T*1_01 ⩽ 780;), where as borders we can define the average temperature between two linearization points.

### 1.3. Model Verification

**Figure 3:**
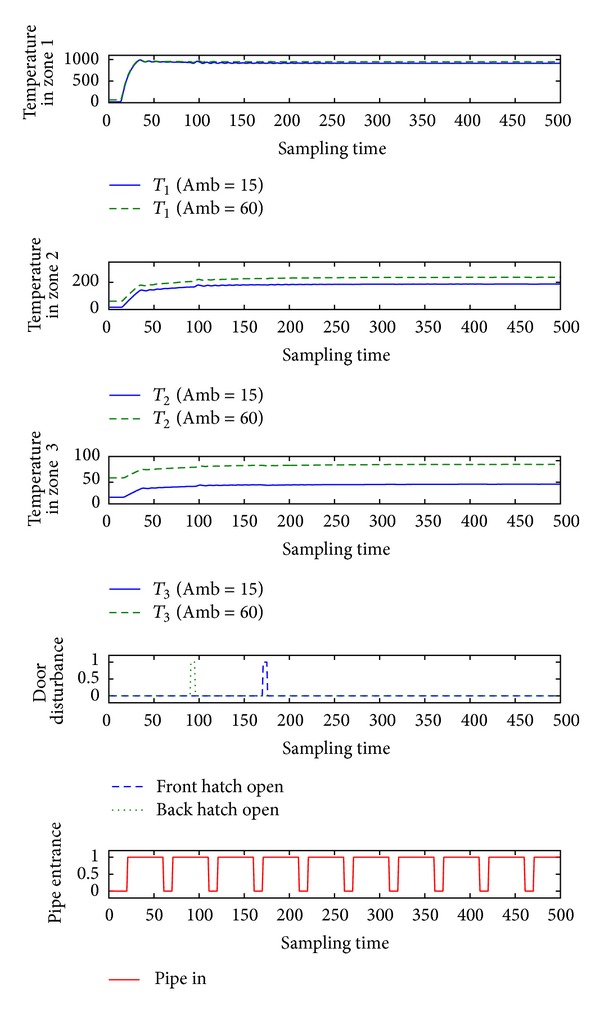
Influence to the zones from control input 1 with respect to the disturbances.

The response of the closed loop hybrid system is satisfactory and stabilizes the temperature in the selected zone to 1000 degrees Celsius, in the zone near to 200, and in the third zone to 40 degrees. A sample closed loop system simulation is shown on Figure 3.

We can conclude that the state that we want to achieve is not feasible but the controller still manages to stabilize near the requested region. This only confirms the quality of the furnace model that acts as the real furnace. The simulations are performed with respect to active disturbances in the systems. The complete results can be found in [9].

## 2. Design of the Controllers

Since we have derived several models for the plant, also we must design controllers for each of the models that will be optimized in a specific way. Besides the standard MPC design and switch MPC design, here we introduce a hybrid MPC and a hybrid multiple-model predictive controller in order to improve the control performance of the industrial furnace. The idea for hybrid control and some results regarding constraints and stability have been explored in details in [10, 11].

The results presented here show a multiple-model MPC of piecewise affine (PWA) system [12] and the design of a complete hybrid MPC for the temperature control of the furnace [13]. The results obtained here clearly justify the use of the hybrid control algorithms over the conventional methods.

For this simulation, we have used one linear MPC, one linear multiple-model MPC, one hybrid MPC, and one hybrid multiple-model MPC. These controllers are tested in equal simulation conditions, and the results are compared.

### 2.1. Controller Synthesis

The optimization problem of linear MPC is known for a long time, and it is not a subject of this paper. For the design of the controller, standard design methods are used. Regarding the hybrid optimization, the problem in control science is relatively new. In this case, we have designed a cost function in the form given in (1) and (2).

Consider

(1)  min⁡{u,δ,z}0N−1J({u,δ,z}0N−1,x(t))=Δ||QxN(x(N ∣ t)−xr)||p+∑k=1N−1||Qx(x(k)−xr)||p +∑k=0N−1||Qu(u(k)−ur)||p+||Qz(z(k ∣ t)−zr)||p +||Qy(y(k ∣ t)−yr)||p,

(2)  s.t. x(0 ∣ t)=x(t),x(k+1 ∣ t)=Ax(k ∣ t)+B1u(k ∣ t)+B2δ(k ∣ t)+B3z(k ∣ t),y(k ∣ t)=Cx(k ∣ t)+D1u(k ∣ t)+D2δ(k ∣ t)+D3z(k ∣ t),E2δ(k ∣ t)+E3z(k ∣ t)≤E1u(k ∣ t)+E4x(k ∣ t)+E5,umin⁡≤u(t+k)≤umax⁡, k∈[0,N−1],xmin⁡≤x(t+k ∣ t)≤xmax⁡, k∈[0,N],ymin⁡≤y(t+k)≤ymax⁡, k∈[0,N−1],Sxx(N ∣ t)≤Tx,

where *N* is the optimal control interval and *x*(*k* | *t*) represents the state predicted at moment *t* + *k* resulting from the input *u*(*t* + *k*). The initial value of the system at time *t* is *x*(0 | *t*) = *x*(*t*); *u*_min⁡_, *u*_max⁡_, *y*_min⁡_, *y*_min⁡_, and *x*_min⁡_, *x*_min⁡_ are hard bound on the inputs, outputs, and states, respectively; and {*x* : *S*_*x*_ *x* ≤ *T*_*x*_} is a final target polyhedral subset of the state-space ℝ^*n*^. In (1), ||*Qx*||_*p*_ = *x*′*Qx* for *p* = 2 and ||*Qx*||_*p*_ = ||*Qx*||_∝_ for *p* = *∞*. In (1) and (2), with *x*(*t*) we represent the continuous states of the system and with *z*(*t*) the discrete states of the system; the inputs are denoted by *u*(*t*) and the outputs by *y*(*t*).

We use the Hybrid Toolbox for Matlab [14] as a design tool for the controller for the high consumption industrial furnace. This toolbox can work with several different types of hybrid system models (e.g., Mixed Logical Dynamical Systems, Piecewise Affine Systems, and Discrete-Time Hybrid Automata) and presents a formal mathematical equivalence between these models. We use HYSDEL to represent the model of the furnace [14, 15]. 

In order to achieve better results, we have divided the temperature domain of the furnace into five sections as presented here: section_1_ = *T*_2_ ∈ [−10,260]; section_2_ = *T*_2_ ∈ [260,520]; section_3_ = *T*_2_ ∈ [520,780]; section_4_ = *T*_2_ ∈ [780,1040]; section_5_ = *T*_2_ ∈ [1040,1300]. For each of the sections, a linearized model for the furnace was derived near the midpoint of the respective section (e.g., for section_4_ the model was linearized near *T*_2_ = 910°Celsius).

MLD hybrid model generated from the HYSDEL file for the multimodel linearized problem has 25 continuous states, 9 inputs (4 continuous, 5 binary), and 3 continuous outputs. The HYSDEL model has 22 continuous auxiliary and 15 binary auxiliary variables. The optimization problem to be solved has 118 mixed-integer linear inequalities. The sampling time of the system is 0.5 minutes. If comparison to the hybrid model of the furnace linearized in one operating point whose HYSDEL representation has only 38 mixed-integer linear inequalities, it is obvious that the complexity of the optimization problem is significantly increased with the introduction of multimodel linearization. This affects the computation time of the optimization algorithm and favors the one point linearization method for implementation if it has satisfactory behavior.

## 3. Simulation Results

In order to compare the quality of the designed controllers, we have designed equivalent simulation conditions for the four algorithms. In this study, we will compare the linear MPC, multiple-model MPC, hybrid MPC, and hybrid multiple-model MPC.

**Figure 4:**
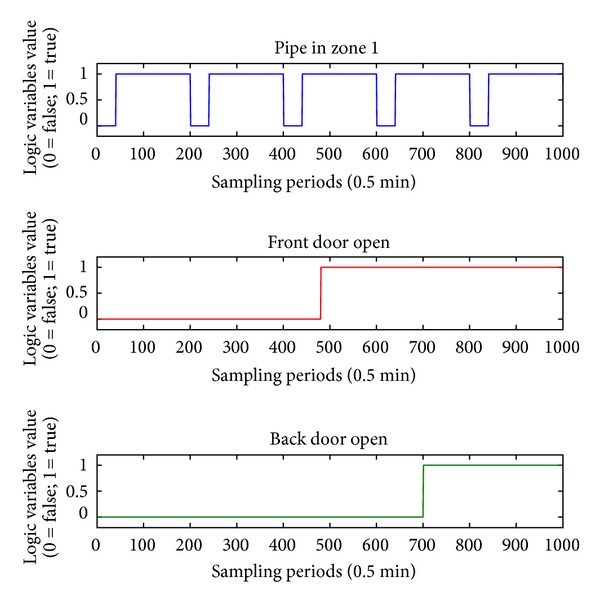
Disturbances during the experiment.

The disturbance signals from the front and the back hatches and the timing of the pipe entering in the first zone of the furnace are graphically represented on Figure 4. In Figure 4, the logic variable for pipes entering zone 1 is presented. The logic variables for zones 2 and 3 have deterministic dependence on this value with fixed delay. In reality, this delay is represented through the line speed of the conveyor driving the pipes in the furnace, but this is to be done in near future. During this simulation, a fixed delay time of 10 minutes between zones is adopted. During the simulation, the continuous disturbance signal has value of 15°Celsius.

**Figure 5:**
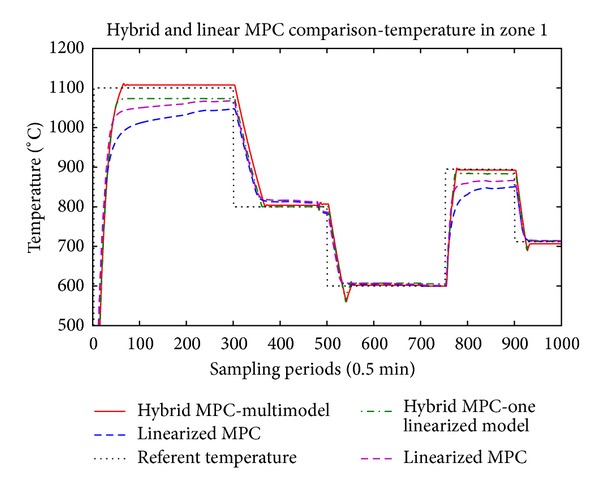
Temperature in the first zone.

**Figure 6:**
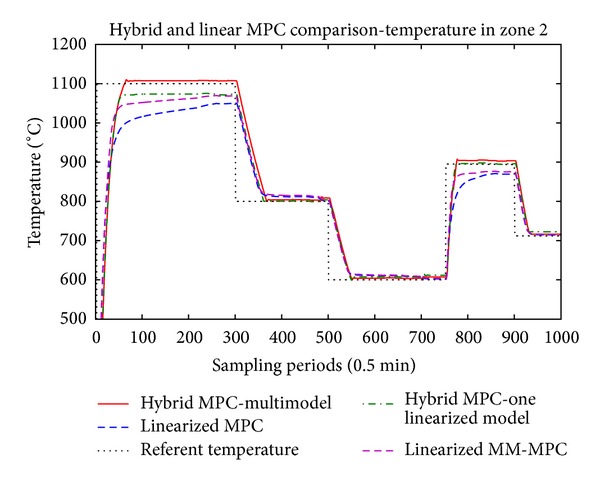
Temperature in the second zone.

**Figure 7:**
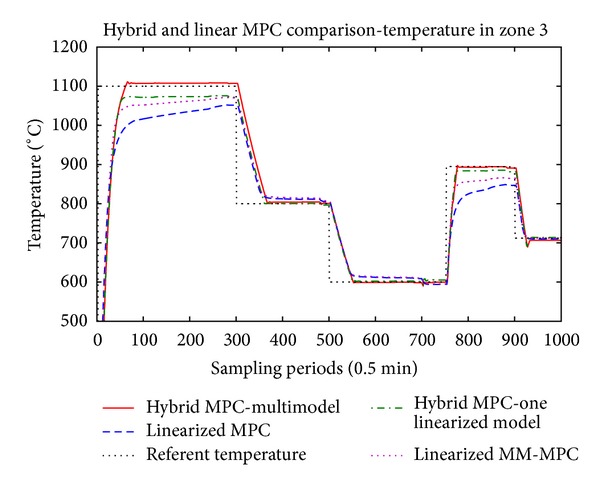
Temperature in the third zone.

**Figure 8:**
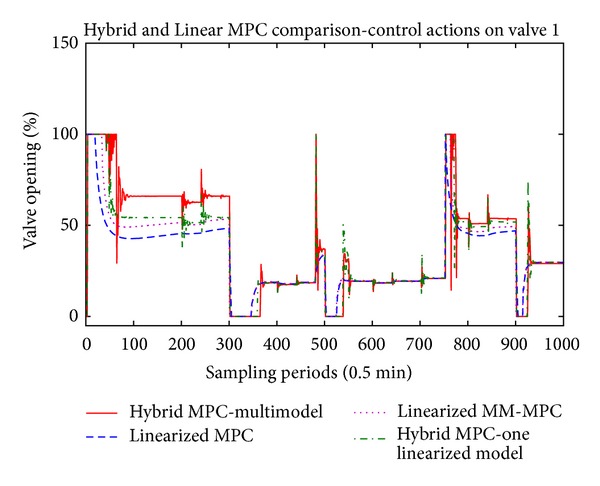
Control actions of the first valve.

**Figure 9:**
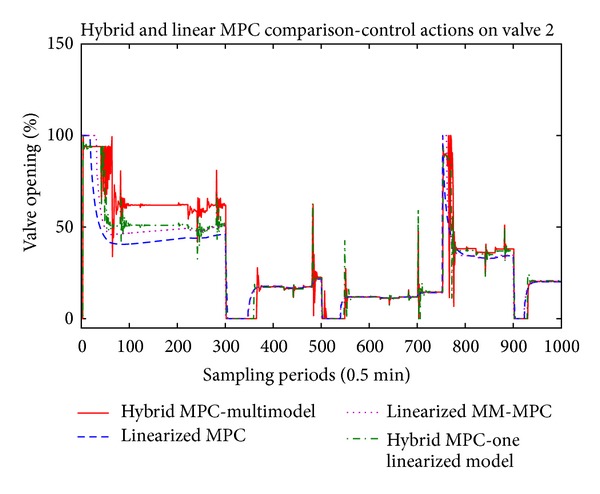
Control actions of the second valve.

**Figure 10:**
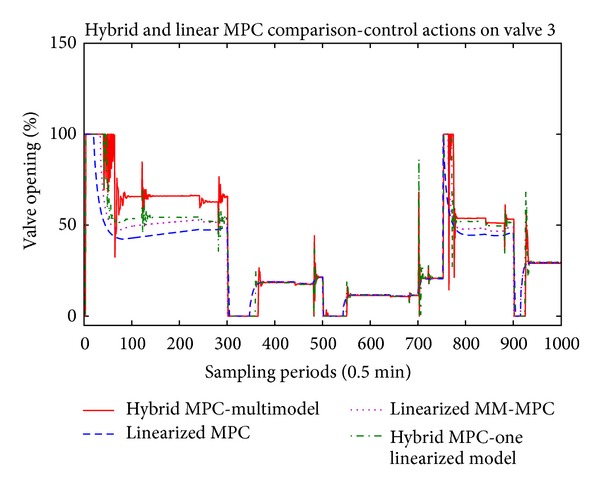
Control actions of the third valve.

The main results are presented in Figures 5, 6, and 7 where the temperatures in the respective zones of the furnace are presented long with the reference signal. The control signals applied to the three control valves, respectively, are presented in Figures 8, 9, and 10. 

From the presented results, it is obvious that introducing the hybrid control approach for high consumption industrial furnace improves the quality of the control. The controller leads the system faster to the referent set-point, and the steady state error is acceptable. The hybrid MPC, one linearized model method, has also satisfactory results. The multiple-model MPC also shows satisfactory results, but there is oscillatory behavior at the reference values. 

Since we have linearized models, the tracking of the referent trajectory is best when it is near the linearization point(s), and as the referent trajectory moves from this point we have bigger error in the control algorithm. This is more expressed in the hybrid controller with only one linearization point, which is linearized near 800° degrees. In this case, it is obvious that output tracks the reference without any problem near this region, but if we have work plans that require a lot of temperature changes throughout the temperature domain of the furnace, the multimodel hybrid approach is to be considered. The previous remark, regarding the performance of the controller near the linearization point, also stands for the multimodel hybrid approach. The difference here is that we have several models, and the difference between the set-point and the active model cannot be very big. Logically, if we introduce more models linearized in different operating points we will increase the performance of the controller, but also we will increase the complexity and the time necessary to perform the optimization. 

Regarding the control signals, on all three figures (Figures 8, 9, and 10) we can note that the hybrid controllers have fast reaction time to the disturbances. When there is new pipe entering in one of the zones of the furnace, the control signal in the respective zone acts towards stabilization of the temperature. Also, we can note that when the furnace is operating near 800° degrees, all three controllers generate the same control value, but if we move far from this central linearization point, the calculated values for the control action differ a lot.

## 4. Conclusion

In order to obtain real measure of the controller's quality, we must compare their performances. Therefore, we have designed a complete test scenario, which defines the conditions during the simulation. Of course we must keep in mind that the proposed algorithms are specific to the problem they address. Anyway, these differences will be explained in detail in this chapter. In Table 1, we present the abbreviations used to specify the algorithms along with the full names.

Discrete automaton for the furnaces states on the first zone.

We have run several simulations for each of the controllers with the same simulation conditions. During these simulations, we have properly tuned up the controllers so we can compare their best performances. Also during the simulations, we have tested the robustness of the controllers by adding small disturbances (opening of the front and back hatches). The parameters chosen for comparison of the controller's performances are the settling time, the average overshoot, the ITAE norm, the time for computation, and the average fuel consumption.

The average values for the results obtained are summarized in Table 2. We must point out that these results are not directly implied by the behavior of the outputs presented in Figures 5–10, but they represent a measure for the total average values of the analyzed parameters.

Regarding the average overshoot, during our simulations we have drawn a general conclusion that by increasing the complexity of the controller, we can reduce the average overshoot (if the controller is properly tuned). The second important notice here is that the multiple-model algorithms generate bigger overshoot than the appropriate single model algorithms. This is a result of the errors in the prediction of the single model controllers. On the other hand, if we look into the ITAE norm we can conclude that the multiple-model algorithms do have better performance than the single model algorithms.

The average computation time increases with the complexity of the algorithm. Anyway, we must mention that the difference in the computation times is not very big; in fact the maximal difference is only 40 ms, which is a very small value for this kind of processes. The fuel consumption increases with the complexity of the controller but does not always guarantee improved control quality.

At the end, we can conclude that introducing techniques for improved control in industrial plants with high consumption will lead to improving the quality of the products. Especially if we incorporate the logical and integer variables in the optimization process we can obtain significantly better results, because the real system do have a need for optimization if hybrid environment.

Before we generalize these results, we must mention that each and every problem in the process industry is different, and the engineer must prepare a detailed study on the problem in order to choose the best algorithm for control. In many cases, the improvements that resulted from implementation of an advanced control technique are very small compared to some classical control methods, such as PID control. 

When we speak of industrial plants with high consumption, these algorithms are very useful since even small improvement in the quality/price criteria could result in increasing the profit of the company. As a result of the implementation of these algorithms, we derive several improvements in the industrial plant control:

- improving the quality of the final products,
- reducing the settling time,
- reducing the fuel consumption,
- improving the robustness of the controller.

It is clear that the companies can easily benefit from the results of the implementation of these algorithms, but, on the other hand, the companies are reluctant to trying new technologies, especially in the countries in development.

Some of the future research possibilities in this area are the complete design of hybrid model of the presented industrial furnace with high consumption. This model should be adaptive, and by proper tuning of its parameters could be easily modified to represent some other industrial furnaces with similar characteristics.

The practical implementation of hybrid controllers in the industry is not at a satisfactory level. Although in theory the hybrid algorithms introduce big improvements, their practical implementations until now are limited to laboratory test-beds or simple processes. 

The issue of robustness of hybrid MPC with respect to the unmodeled disturbances in the system should also be addressed. Since in all industrial plants we have significant number of not modeled disturbances, research in this direction could result with significant improvements.

## Figures and Tables

**Table 1:** Abbreviations and full meaning of the algorithm's names.

Abbreviations	Full meaning
MPC	Model predictive control (usually linearized)
MM-MPC	Multiple-model model predictive control
Hybrid MPC	Hybrid model predictive control
Hybrid MM-MPC	Hybrid multiple-model model predictive control

**Table 2:** Comparisons of the proposed MPC algorithms.

Algorithm	Settling time	Average overshoot	ITAE norm	Computation time	Consumption
MPC	*≈*110	10%	160,410	*≈*100 ms	89,887
MM-MPC	*≈*90	6%	115,357	*≈*110 ms	97,328
Hybrid MPC	*≈*35	3%	111,770	*≈*130 ms	100,880
Hybrid MM-MPC	*≈*35	5%	96,736	*≈*140 ms	110,310

## References

[1] Rhine J, Tucker R (1991). *Modeling of Gas-Fired Furnaces and Boilers*.

[2] Dimirovski G, Dourado A, Gough N On learning control in industrial furnaces and boilers.

[3] Stojanovski G, Stankovski M Advanced industrial control using fuzzy-model predictive control on a tunnel kiln brick production.

[4] Lee JH (2011). Model predictive control: review of the three decades of development. *International Journal of Control, Automation and Systems*.

[5] Mayne DQ, Rawlings JB, Rao CV, Scokaert POM (2000). Constrained model predictive control: stability and optimality. *Automatica*.

[6] Camacho E, Bordons C (2004). *Model Predictive Control*.

[7] Maciejowski J (2002). *Predictive Control with Constraints*.

[8] Stankovski M (1997). *Non-conventional control of industrial energy processes in large heating furnaces [Ph.D. dissertation]*.

[9] Stojanovski G, Stankovski M Hybrid modeling of industrial furnace using HYSDEL.

[10] Bemporad A, Morari M (1999). Control of systems integrating logic, dynamics, and constraints. *Automatica*.

[11] Bemporad A, Heemels WPMH, De Schutter B (2002). On hybrid systems and closed-loop MPC systems. *IEEE Transactions on Automatic Control*.

[12] Stojanovski G, Stankovski M, Dimirovski G (2010). Multiple-model model predictive control for high consumption industrial furnaces. *Facta Universitatis, Series: Automatic Control and Robotics*.

[13] Stojanovski G, Stankovski M (2012). A hybrid system approach for high consumption industrial furnace control. *Intelligent Control and Automation*.

[14] Torrisi FD, Bemporad A (2004). HYSDEL—a tool for generating computational hybrid models for analysis and synthesis problems. *IEEE Transactions on Control Systems Technology*.

[15] Bemporad A Hybrid Toolbox—User Guide. http://cse.lab.imtlucca.it/~bemporad/hybrid/toolbox/.

